# Supplementary material for: LRRC8/VRAC anion channels enhance β-cell glucose sensing and insulin secretion
Source: Nat Commun. 2018 May 17;9:1974. doi: 10.1038/s41467-018-04353-y (PMC5958052; doi:10.1038/s41467-018-04353-y)
Supplement: Supplementary file 1 — Supplementary Information [file 41467_2018_4353_MOESM1_ESM.pdf]

Supplementary Information for

'LRRC8/VRAC anion channels enhance β-cell glucose sensing and insulin secretion'

by Stuhlmann, Planells-Cases and Jentsch

Supplementary Figures

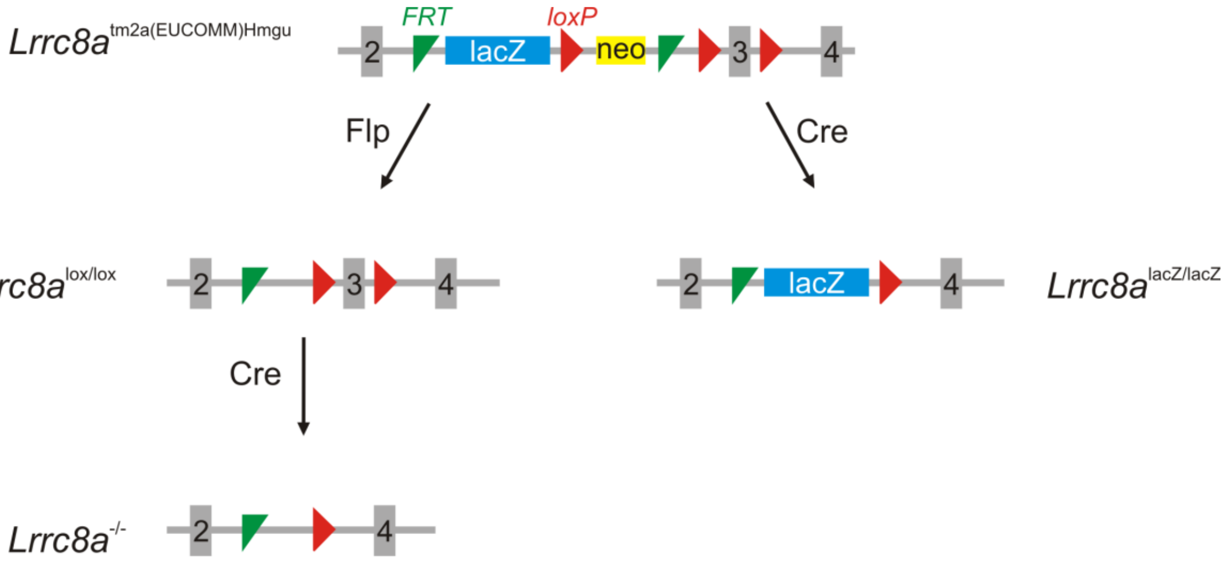

Supplementary Figure 1. Targeting of the *Lrrc8a* gene. The targeted *Lrrc8a* allele (*Lrrc8a*$^{\text{tm2a(EUCOMM)Hmgu}}$) generated by EUCOMM (European Conditional Mouse Mutagenesis Program) in embryonic stem cells contains the bacterial *lacZ* gene (*Escherichia coli*) and a neomycin cassette (neo) flanked by *FRT*-sites, as well as additional *loxP*-sites flanking exon 3. Exon 3 codes for the first 719 amino acids of the protein. A pure floxed allele (*Lrrc8a*$^{\text{lox/lox}}$) can be created by flippase (Flp) recombinase expression in mice carrying the targeted *Lrrc8a*$^{\text{tm2a(EUCOMM)Hmgu}}$ allele. Expression of the Cre-recombinase, which might be driven by a cell-type specific promoter as in the present work, disrupts the *Lrrc8a* gene (*Lrrc8a*$^{-/-}$). Cre expression without prior Flp expression in heterozygous *Lrrc8a*$^{+/\text{tm2a(EUCOMM)Hmgu}}$ mice generates a reporter mouse (*Lrrc8a*$^{+/\text{lacZ}}$) (which, in contrast to *Lrrc8a*$^{-/-}$ mice[1], is viable because only one *Lrrc8a* allele is disrupted) and which can be used to examine *Lrrc8a* expression by β-galactosidase staining. Exons are depicted as grey boxes. Schematic drawing is not to scale.

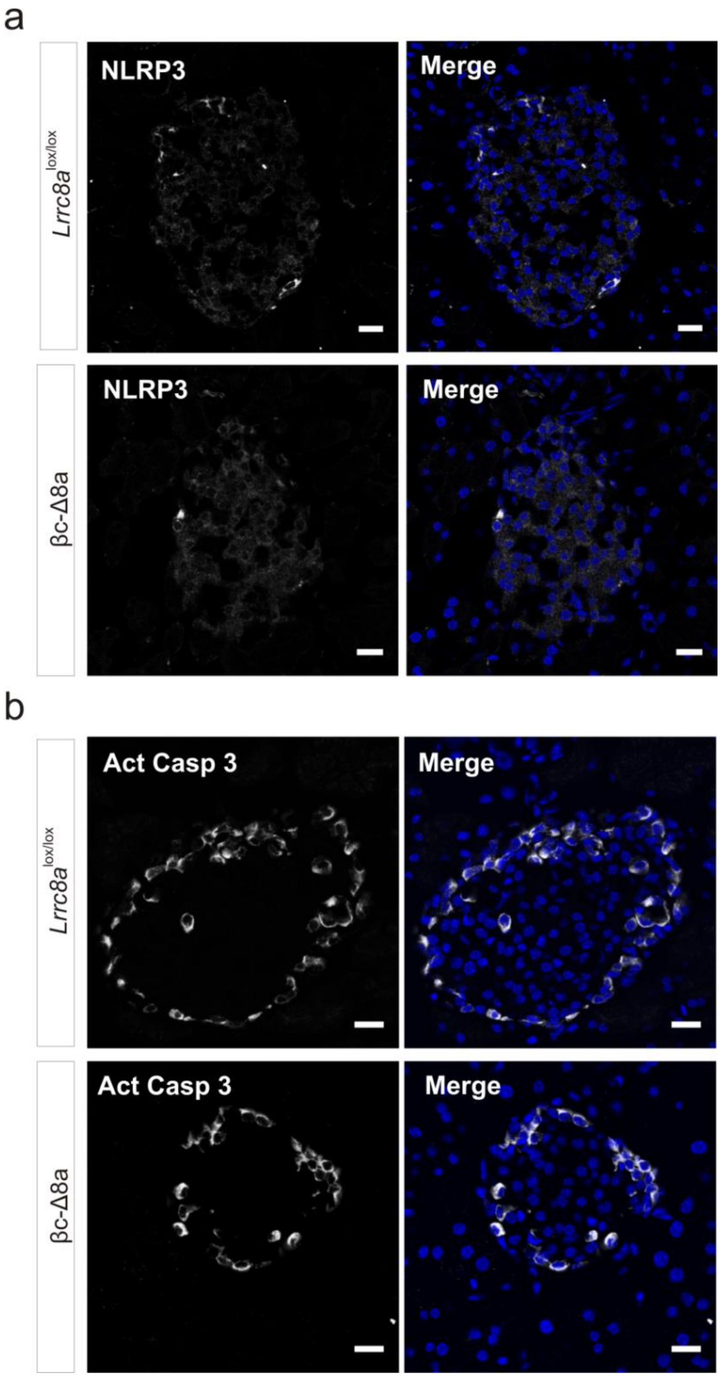

Supplementary Figure 2 No indication for differences in inflammasome or caspase activation between the genotypes. a Immunofluorescent labeling for the NLRP3 inflammasome component (grey) together with nuclear staining with DAPI (blue). Representative images from pancreatic sections from 2 paired *Lrrc8a*$^{lox/lox}$ and βc-Δ8a animals. **b** Immunofluorescence staining using an an antibody against active caspase 3 (grey) together with nuclear staining using DAPI (blue). Representative images from pancreatic sections from 2 paired animals per genotype. Similar caspase 3 labeling was found in islets from healthy B6 mice [2]. Scale bars, 20 µm.

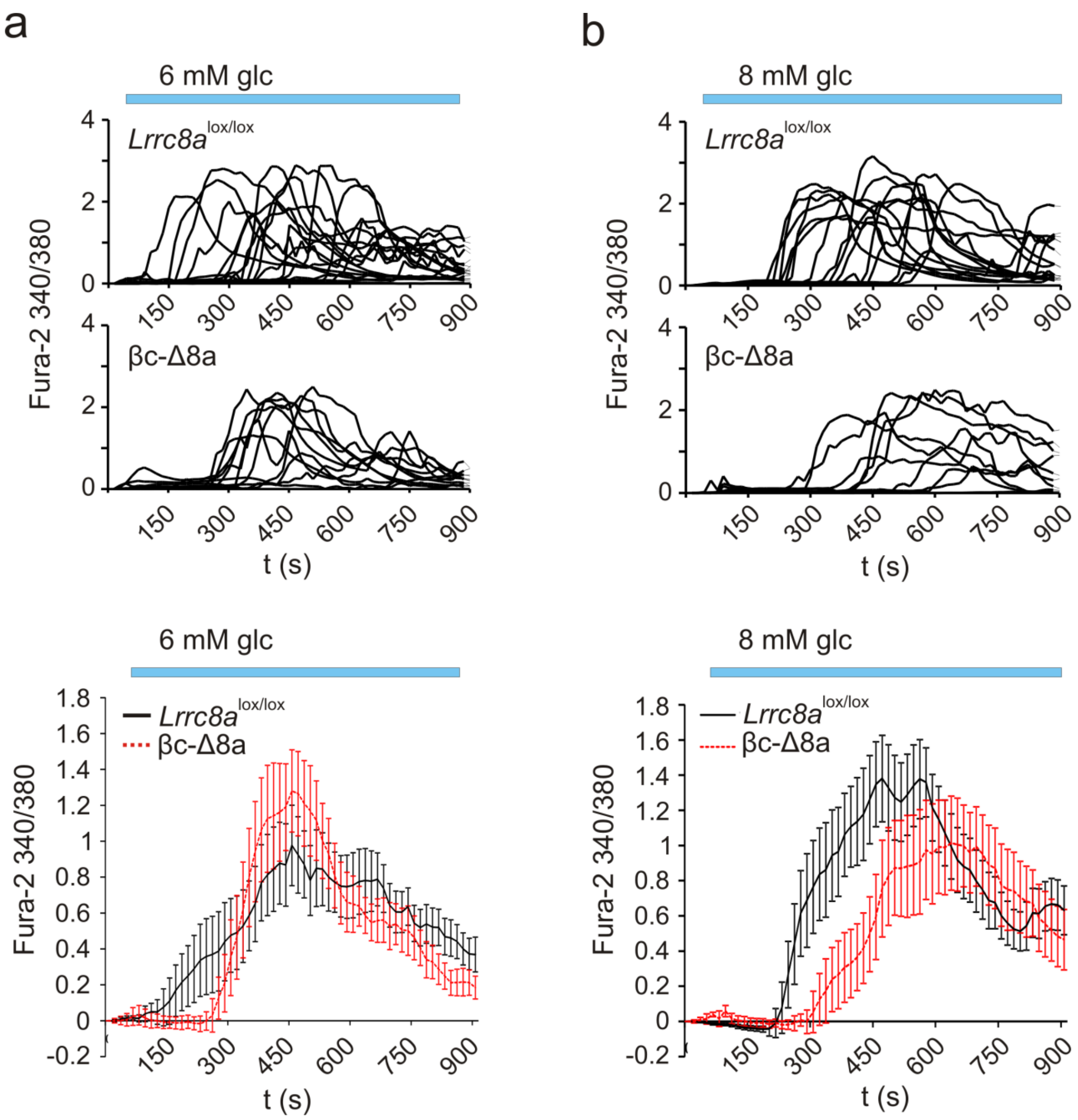

Supplementary Figure 3. Intracellular Ca^{2+} response in *Lrrc8a*$^{lox/lox}$ and βc-Δ8a cells to moderate increases in glucose concentrations. **a**, **b** Individual traces (upper panels) and the mean ratio (lower curves) of Fura-2 fluorescence ratios following stimulation with 6 (**a**) or 8 (**b**) mM glucose at t = 60 s, following preincubation with 3 mM glucose. Mean values ± SEM, **a** n=18 and n=11 for *Lrrc8a*$^{lox/lox}$ and βc-Δ8a β-cells, respectively, **b** n=15 and n=11 for *Lrrc8a*$^{lox/lox}$ and βc-Δ8a β-cells, respectively.

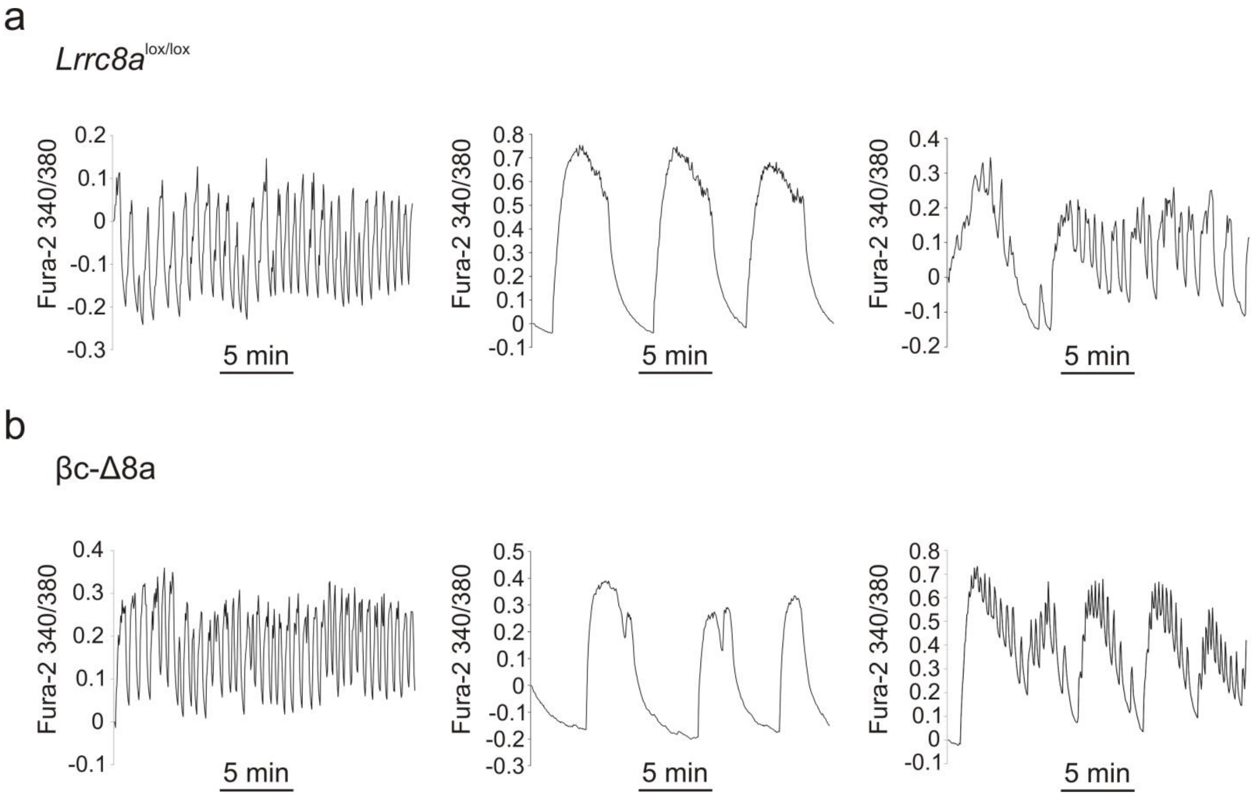

Supplementary Figure 4. Glucose-induced Ca^{2+} oscillations of intact islets from *Lrrc8a*$^{lox/lox}$ and βc-Δ8a mice. a, b Individual traces of Fura-2 fluorescence ratios of *Lrrc8a*$^{lox/lox}$ (**a**) and βc-Δ8a (**b**) islets following stimulation with 10 mM glucose. 10 mM glucose was added 30 min before the experiments and was present throughout the measurements. Islets of both genotypes displayed different Ca^{2+} oscillation patterns, as previously described[3]. 17 *Lrrc8a*$^{lox/lox}$ islets and 18 βc-Δ8a islets from 4 different mice per genotype were analyzed. No obvious differences between the genotypes were found.

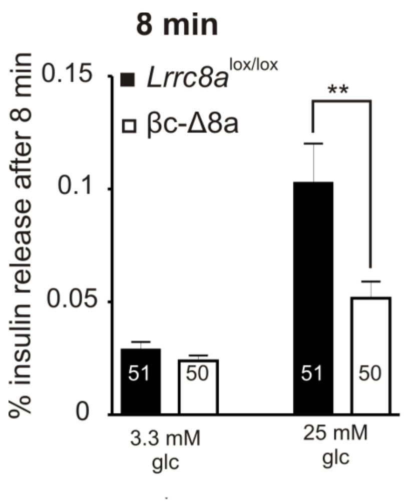

Supplementary Figure 5. Insulin secretion of isolated islets in the presence of 3.3 or stimulated by 25 mM glucose (during the first 8 minutes). Number of islets (from 4 mice per genotype) is indicated in bars. **, $p<0.01$ (one-way ANOVA, Tukey's test).

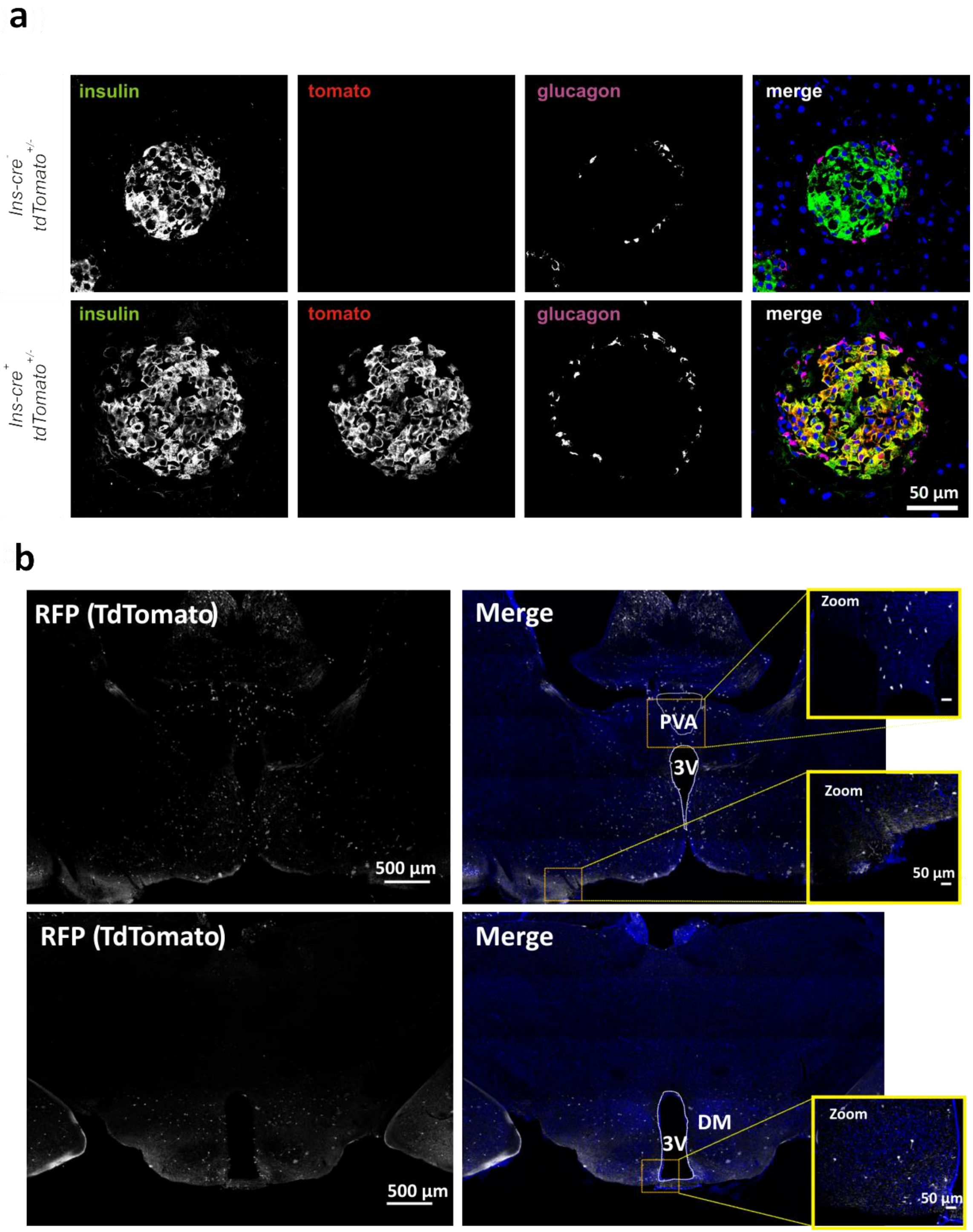

Supplementary Figure 6. Specificity and efficiency of the β-cell specific Cre line. Ins2-Cre (*Tg(Ins2-cre)23Herr*) mice[4] were crossed to reporter mice (B6.Cg-*Gt(ROSA)26Sor*$^{tm9(CAG\text{-}tdTomato)Hze}$/J) expressing tdTomato upon Cre-recombinase expression[5]. **a**. Pancreatic sections immunostained with RFP antibody to detect TdTomato (stained red in merged pictures at right). TdTomato was not detected in the absence of the Cre-recombinase (upper panels), but expressed in virtually all β-cells (identified by insulin staining, green in the merged pictures at right), but not in glucagon-positive α-cells (stained in magenta)

nor in other cells. Nuclei were identified by blue DAPI staining. **b.** Ins2-Cre recombinase activity in the brain is scarce and not restricted to specific regions of the brain or of the hypothalamus. Images of two individual coronal brain slices show an anterior (upper panel) and a posterior (lower panel) view of the hypothalamus. 50 µm thick free floating slices were stained with RFP antibody (white) and nuclei counterstained with DAPI (blue). Zoom images in left column display detailed staining in different brain areas.3V, third ventricle; PVA= Paraventricular thalamic nucleus; DM= Dorsomedial hypothalamic nuclei.

a

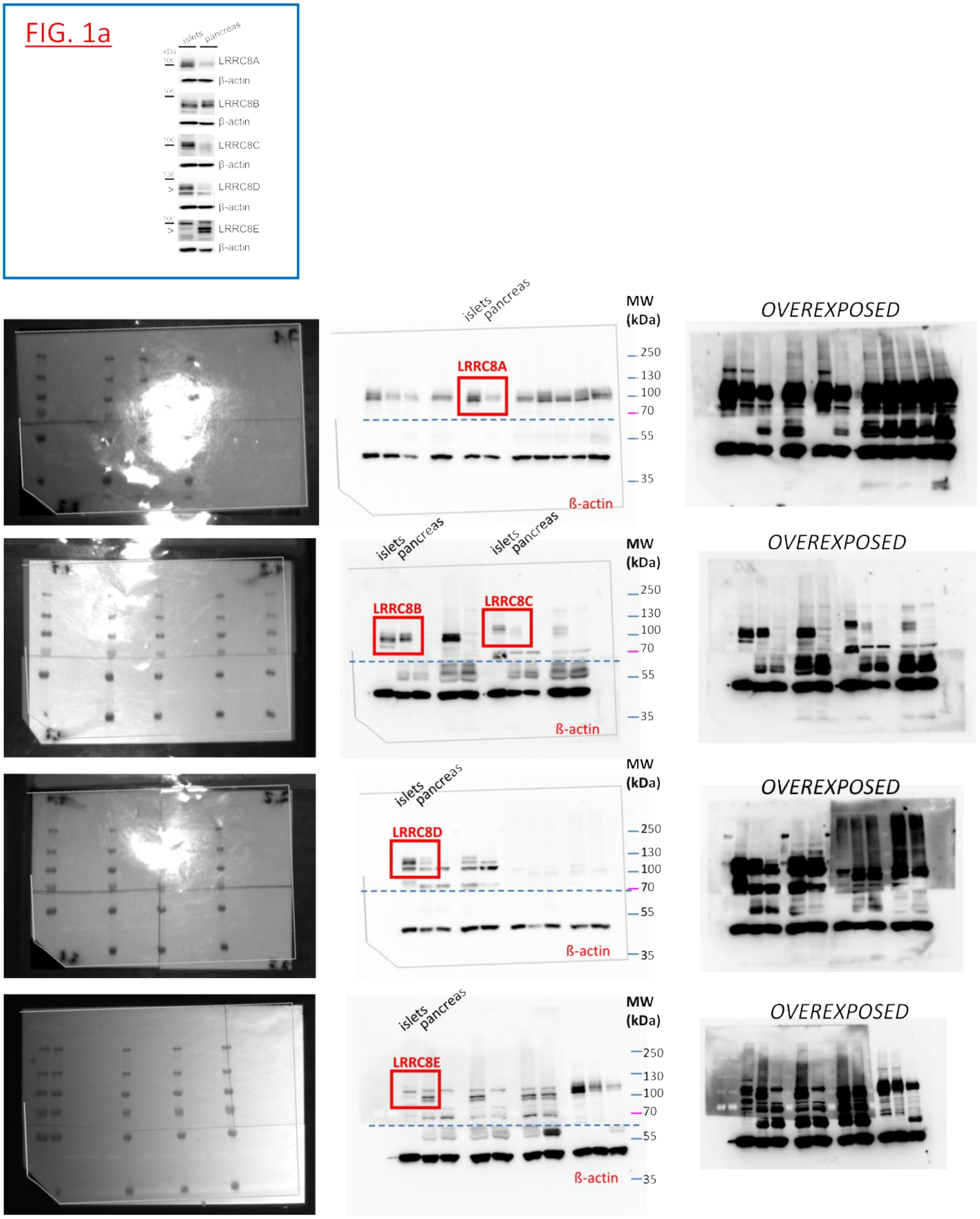

(Supplementary Fig 7, continued)

b

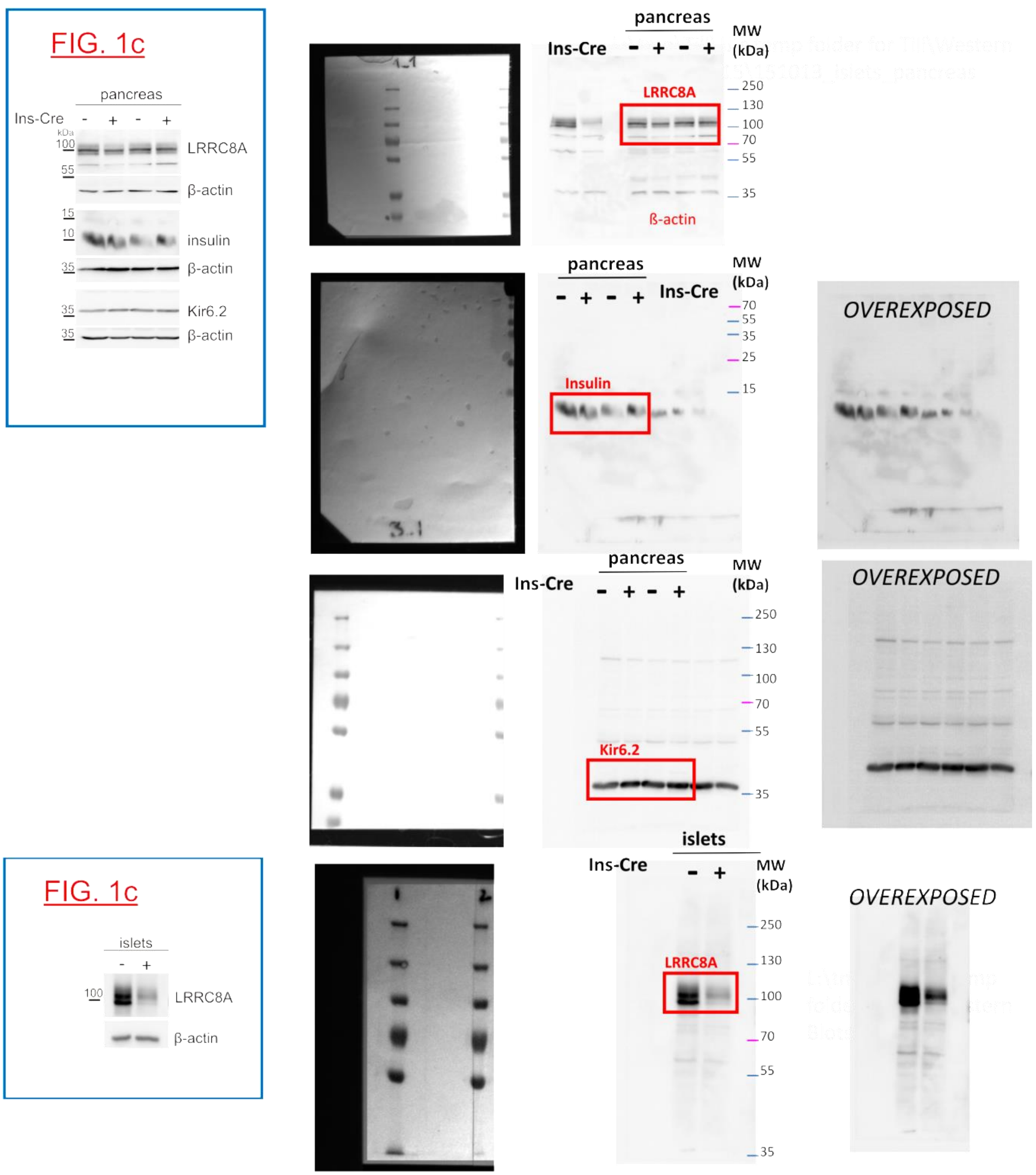

Supplementary Figure 7. Uncropped images of Western blots displayed in Fig. 1. a, blots belonging to panel 1a. **b,** blots belonging to panel 1c. Blots shown in main article are boxed in blue. Photographs of membranes reveal stained marker proteins (left), and Western blots at right are shown also after overexposure to better reveal the form of membranes for comparison with the photographs.

References

1 Kumar, L. *et al.* Leucine-rich repeat containing 8A (LRRC8A) is essential for T lymphocyte development and function. *The Journal of experimental medicine* **211**, 929-942 (2014).

2 Ning, Y. *et al.* Ranolazine increases β-cell survival and improves glucose homeostasis in low-dose streptozotocin-induced diabetes in mice. *J Pharmacol Exp Ther* **337**, 50-58 (2011).

3 Bertram, R., Sherman, A. & Satin, L. S. Electrical bursting, calcium oscillations, and synchronization of pancreatic islets. *Advances in experimental medicine and biology* **654**, 261-279 (2010).

4 Herrera, P. L. Adult insulin- and glucagon-producing cells differentiate from two independent cell lineages. *Development* **127**, 2317-2322. (2000).

5 Madisen, L. *et al.* A robust and high-throughput Cre reporting and characterization system for the whole mouse brain. *Nat Neurosci* **13**, 133-140 (2010).
